# The impact of self-reported exposure to whole-body-vibrations on the risk of disability pension among men: a 15 year prospective study

**DOI:** 10.1186/1471-2458-10-305

**Published:** 2010-06-03

**Authors:** Finn Tüchsen, Helene Feveile, Karl B Christensen, Niklas Krause

**Affiliations:** 1 Department of Epidemiology and surveillance, The National Research Centre for Working Environment, Lersø Parkallé 105, DK-2100 Copenhagen Ø, Denmark; 2 Department of Biostatistics, Institute of Public Health, Øster Farimagsgade 5, DK-1014 Copenhagen K, University of Copenhagen, Copenhagen, Denmark; 3 Department of medicine, University of California at San Francisco, 1301 South 46th St. Building 163, Richmond, CA 94804, USA

## Abstract

**Background:**

Whole-body-vibrations are often associated with adverse health effect but the long term effects are less known. This study investigates the association between occupational exposures to whole-body vibrations, and subsequent transition to disability pension.

**Methods:**

A total of 4215 male employees were followed up for subsequent disability pension retirement. Exposure to whole-body-vibration was self-reported while new cases of disability pension were retrieved from a national register.

**Results:**

The hazard ratio (HR) for disability pension retirement among men exposed to whole-body-vibrations was 1.61 (95% confidence interval (CI) 1.07-2.40) after adjustment for age, smoking habits, BMI, physical job demands and awkward work postures. In our model, with the available explanatory variables, 5.6% of the male disability pension cases were attributable to whole-body-vibrations.

**Conclusions:**

Exposure to whole-body-vibrations predicts subsequent disability pension retirement. Continued reduction of whole-body-vibrations may reduce the number of new cases of disability pension.

## Background

Disability retirement is a burden and a loss of opportunities for individuals, their family, employers and the society at large. It also causes loss of esteem and self-esteem. The elderly fraction of the population is growing and the young and productive part of the population is diminishing. Shortage of labour is a growing problem in the health care sector, in education, in cleaning etc. This has increased the interest in reducing early age disability pension and to introduce more flexibility to the disability pension rules allowing part time work and re-entrance into the work force for those who earlier had to face an often marginalized life as retiree.

Disability retirement rates differ between industries and it has been estimated that roughly 38-40% can be attributed to non-optimum work environment [1]. Albertsen and colleges [2] provided an overview of demographic, lifestyle and work environmental risk factors for disability pension. Age, gender, smoking habits, Body Mass Index (BMI), physical job demands and awkward work postures along with various aspects of the psychosocial working environment were mentioned. The present study is based on an expansion of the data and a refinement of the methods employed in that study and investigates the association between whole-body-vibration (WBV) and subsequent transition to disability pension.

Low back pain hits almost everyone sometime during life [3] and it is a major cause of disability pension. Worldwide, 37% of low back pain was attributed to occupation, especially those involving lifting and WBV [4].

Exposures to WBV are common among drivers of cars, vans, lorries, forklift trucks, tractors [5], all-terrain vehicles [6] including armed forces armoured vehicles [7], buses and loaders [8] as well as earth moving machinery [9] and cranes [10].

WBV has been identified as a cause of musculoskeletal disorders such as low back pain [11-13], and may play a role for lower leg or calf pain, and ankle or foot pain [14]. Long sickness absence spells are one of the consequences [15]. Another less studied topic is permanent disability pension [5], the outcome of interest for this investigation.

The aims of this study are to estimate the relative risk of disability pension due to exposure to WBV and to estimate the fraction of disabilities that can be attributed to WBV.

## Methods

This study is based on a merger of survey data about the work environment from the Danish Work Environment Cohort Study (DWECS) and information about granted disability pensions from the national register DREAM (a Danish acronym for The Register-based Evaluation of Marginalisation). DREAM is an administrative register holding weekly information of social transfer payments for all inhabitants in Denmark [16].

### Baseline

DWECS is a representative national survey of work environment and health conducted every fifth year [17]. DWECS has a split panel design: On 1^st ^October 1990 a simple random sample of inhabitants of Denmark between 18 and 59 years of age was drawn from the Danish centralised civil register. On 1^st ^October 1995 additional simple random samples of immigrants (in the previous 5 years) and young persons (between 18 and 22 years of age) were drawn to update the 1990-panel with respect to immigration and ageing. The sizes of the immigrant sample and the young sample were determined by proportional allocation (e.g. immigrants/young persons constitute the same proportion in the total 1995-sample as in the 1995-population). On 1^st ^October 2000 additional simple random samples of immigrants and young persons were drawn according to the same procedure as in 1995, supplementing the 1995-panel to reflect the 2000-population. Persons sampled for one of the surveys were automatically included in the following surveys, and approached for these survey irrespectively of whether they participated in previous surveys or not. A figure illustrating the composition of the samples for the 1990, 1995 and 2000 surveys can be found in Burr et al [17].

In each of the three surveys, data was collected using primarily telephone interviews with personal interviewing as second alternative (6 - 12% of the respondents), and respondents who had been employees within two months prior to the interview were interviewed about working conditions, occupational exposures and health behaviours.

The 1990-sample consisted of 9,653 people, of which 8,664 (90%) participated, 5,701 of these were employees. The total 1995-sample, that is the 1990-sample along with the immigrant and the young sample from 1995, consisted of 10,702 people, of which 8,583 (80%) participated, 5,369 of these were employees. The total 2000-sample, that is the total 1995-sample along with the immigrant and the young sample from 2000, consisted of 11,437 people, of which 8,583 (75%) participated, 5,366 of these were employees. In total, 8,475 respondents were employees in at least one of the surveys and thereby eligible for analysis; 4,288 men and 4,187 women. Since only 1.3% of the female employees were exposed to WBV in each of the three waves of DWECS and only one exposed female employee was granted disability pension in the follow-up period, the present study included only male employees; 1,549 who participated in one interview, 1,275 who participated in two interviews and 1,464 who participated in three interviews.

### Follow-up

Persons were followed in the DREAM register from the time of their first DWECS interview and were censored, at the time of their 60th birthday, emigration, death, or end of follow-up (June 18th 2006). Those participating in a later DWECS interview were followed again from the time of the later interview with updated exposure and confounder information. Those not participating in later DWECS interviews were followed with the existing exposure and confounder information. In total 4215 male employees without missing data who were under the age of 60 at the time of interview were included in this study.

### Outcome definition

A male employee was defined as a case at the first week he receive disability pension during follow-up. Since DREAM is an administrative register and has developed over time, not all registrations are tailored to our design. Starting in the year 2000, all transitions to disability pension were registered. Before 2000 the registration is complete only if the person concerned still receive disability pension in 2000. Thus registrations for persons who were granted disability pension during the follow-up period but passed on to old-age pension, emigrated or died before the year 2000, might be incomplete in the sense that transitions to disability pension were not registered. Disability pensioners in light jobs (in Danish "skånejob") were not defined as cases since DREAM-registration of these started in 2000.

Information about the underlying diagnosis for disability pension was not available and therefore not considered.

### Exposure and covariate assessment

The exposure question was identical in all three waves of DWECS: "Are you exposed to vibrations affecting the entire body (e.g. from a tractor, truck or other machine)?" The question had 6 response categories: "Almost all working hours", "3/4 of working hours", "1/2 of working hours", "1/4 of working hours", "Seldom" and "Never". Those reporting exposure 1/4 of working hours or more were categorized as exposed, whereas those responding "Seldom" or "Never" were categorized as unexposed.

BMI was calculated from self-reported weight and height and categorized according to the standard classification of the National Institutes of Health (BMI < 18.5, underweight; BMI 18.5-24.9, normal; BMI 25-29.9, overweight; BMI ≥ 30, obese). The population was divided into heavy smokers (15 cigarettes or more per day), moderate smokers (less than 15 cigarettes per day), ex-smokers, and non-smokers. Physical job demands and awkward work postures were assessed with questions on physically hard work, working with hands above the shoulders, and working in a squatting or kneeling position. These three questions had the same 6 response categories as the exposure question. Those responding "Seldom" or "Never" where categorized as unexposed, whereas those responding between "1/4 of working hours" and "Almost all working hours" were categorized as exposed. Only questions appearing in all three waves of DWECS were available for this analysis limiting the number of covariates that could be included in the analysis.

### Statistics

To estimate the independent effect of WBV on the incidence of disability pension, Cox proportional hazards models were used adjusting for age, smoking, BMI, physical job demands and awkward work postures. Both exposure and covariates were entered in the models as time-dependent. Age was a continuous variable entered as a linear effect and the other covariates were categorical (nominal). Only observations with complete data on exposure as well as covariates from the baseline interview were included in the analysis.

In order to assess the robustness of the results towards the incompleteness of the disability pension registration before the year 2000 (cf. the "method" section), the analysis was repeated including only male employees below the age of 50, since they will not reach the censoring age of 60 by the end of 2000.

The fraction of transition to disability pension attributable to WBV was estimated from the time at risk and the estimated hazard ratio from the most extended model using the Miettinen formula [18].

All analyses were performed using the SAS system version 9.1. The Cox proportional hazards models were fitted using the PROC PHREG procedure.

For descriptive purposes the exposure was summarized on job and industry level. Jobs were classified by means of the Danish version of the International Standard Classification of Occupations (ISCO) [19]. Industries were classified according to the Danish version of NACE [20].

## Results

Total follow-up time of the 4,215 male employees included in this study constituted 60,068 person years at risk. During the follow-up 188 of these employees were granted disability pension.

Of the 2,947 male employees contributing from the 1990-survey, 290 reported being exposed to WBV and 190 of these contributed with updated information from the 1995-survey. Among those 190, 79 persons reported WBV in 1995. Of the 2,691 male employees contributing from the 1995-survey, 262 reported being exposed to WBV and 171 contributed with updated information from the 2000-survey. Among those, 60 persons reported WBV in 2000.

Table 1 shows the distribution of the exposures and the covariates among the male participants in each of the survey years.

**Table 1:** Distribution of the exposure and covariates considered in the analysis.

Variable	Category	1990 (N = 2947)	1995 (N = 2691)	2000 (N = 2566)
WBV		9.8	9.7	9.2
Age (mean)		37.4	37.9	39.2
Smoking	Heavy smokers	32.4	29.1	27.5
	Moderate smokers	17.3	14.8	12.7
	Ex-smokers	18.9	20.6	22.4
	Never-smoked	31.5	35.6	37.5
BMI	Underweight	0.6	0.7	0.5
	Normal	61.0	56.0	51.3
	Overweight	32.6	36.4	38.8
	Obese	5.8	6.9	9.4
Physically hard work		16.8	16.1	13.9
Working with hands above shoulders		17.3	13.9	17.0
Squatting or kneeling		17.7	12.9	14.5

Percentage in each survey.

Table 2 shows estimated hazard ratios for disability pension. Exposure to WBV was associated significantly with disability pension among male employees. Controlling for age, smoking habits, BMI, physical job demands and awkward work postures reduced effects slightly from an excess risk of 89 percent to 61 percent but the associations remained statistically significant.

**Table 2:** Estimated hazard ratios (HR) for transition to disability pension among male employees in Denmark 1990-2006.

		*Person years at risk*	*Events*	HR* (95% CI)	HR** (95% CI)	HR*** (95% CI)
WBV	Yes	5923	30	**1.89 (1.28-2.80)**	**1.71 (1.15-2.54)**	**1.61 (1.07-2.40)**
	No	54145	158	1.00	1.00	1.00
Age	+ 1 year			1.07	1.07	1.07
Smoking	Heavy smokers	18517	94		2.05	2.00
	Moderate smokers	9325	26		1.20	1.21
	Ex-smokers	11517	28		0.90	0.90
	Non-smokers	20709	40		1.00	1.00
BMI	Underweight	425	3		3.21	3.06
	Normal	34306	102		1.00	1.00
	Overweight	21216	62		0.77	0.77
	Obese	4122	21		1.15	1.14
Physically hard work	Yes	10042	42			1.52
	No	50027	146			1.00
Working with hands above shoulders	Yes	9916	28			0.76
	No	50153	160			1.00
Squatting or kneeling	Yes	9476	34			1.19
	No	50592	154			1.00

	Total	60068	188			

For the exposure, WBV, 95% confidence intervals (CI) are included.

*) Adjusted for age.

**) Adjusted for age, smoking habits and BMI.

***) Adjusted for age, smoking habits, BMI, physical job demands and awkward work postures.

The analysis including only male employees below the age of 50 comprised 49.466 person years at risk and 120 transitions to disability pension. The estimated HR for disability pension retirement among men exposed to WBV was 1.77 (95% CI 1.09-2.86) when controlling for age, 1.66 (95% CI 1.02-2.69) when also controlling for smoking habits and BMI and 1.44 (95% CI 0.88-2.38) when also controlling for physical demands and body postures.

We found the highest proportions with WBV exposure were found among agricultural workers and store and dock workers. There were decreasing proportions of exposed workers in the traditionally high exposed groups. (Data not shown). The percentage of exposed workers in the entire workforce had, however, only gone down from 9.8% to 8.9% between the years 1990 and 2000.

Table 3 shows the change in the percentage reporting WBV within broad industrial groups. In 1990 50% of the employees in agriculture, horticultures and forestry were exposed to WBV but only 28.6% in 2000.

**Table 3:** Industry groups, number of male employees, number and percentage exposed to WBV.

Industry	1990			1995			2000		
	
	N	Exposed	%	N	Exposed	%	N	Exposed	%
Agriculture, horticulture and forestry	72	36	50.0	69	25	36.2	49	14	28.6
Fishing	10	4	40.0	3	2	66.7	4	1	25.0
Mining and quarrying	11	2	18.2	5	1	20.0	7	0	0.0
Manufacturing	865	84	9.7	831	82	9.9	745	73	9.8
Electricity, gas, heating and water supply	32	0	0.0	33	3	9.1	26	1	3.8
Construction	320	26	8.1	262	35	13.4	266	25	9.4
Trade and repair works	396	24	6.1	372	24	6.5	321	23	7.2
Hotel and restaurants	47	1	2.1	45	0	0.0	41	2	4.9
Transport, storage and communication	305	69	22.6	265	62	23.4	253	50	19.8
Financial intermediation, insurance etc.	87	0	0.0	65	1	1.5	71	0	0.0
Letting and sale of real estate, business activities, etc.	186	6	3.2	201	5	2.5	265	11	4.2
Public administration and defense, etc.	296	40	13.5	205	22	10.7	207	27	13.0
Education	194	4	2.1	185	4	2.2	176	2	1.1
Human health activities, social welfare institutions etc.	141	0	0.0	160	3	1.9	158	5	3.2
Organizations, cultural and sporting activities	86	4	4.7	100	7	7.0	97	5	5.2
Private housekeeping with employees	2	0	0.0	2	0	0.0	0	0	0.0
International organizations	4	0	0.0	2	0	0.0	21	3	14.3

Total	3097	303	9.8	2846	278	9.8	2714	242	8.9

In our model, with the available explanatory variables, 5.6% of the disability pension retirement pension cases were attributable to WBV.

## Discussion

This study identified exposure to WBV as a risk factor for future disability pension among men.

The strength of this study is the prospective design and the five year follow-up period of exposure and confounders for each of three survey waves, thus approaching time-varying exposure and confounder assessment. An important limitation of our study is self-report of exposure. However, in contrast to other work exposures (e.g. time spent bending or twisting the back) it is relatively easy to remember time spent driving or working on vibrating platforms etc. Our self-report measure of WBV was further limited to duration of exposure as no information was obtained regarding the intensity or peak exposures. While objective measurement of WBV would be preferable, its application is not feasible in a population-based epidemiological study because of prohibitive costs and logistics, especially if repeat measures are planned.

It has been estimated that occupational lifting causes twice as many cases of low-back pain than WBV [13]. Information about lifting was not available in the present study; however, adjustment for physical job demands did not substantially reduce the effect sizes. The effects were large so that confounding due to heavy lifting is extremely unlikely to explain the results.

The outcome measure is part of the national DREAM register. Registrations for persons who are granted disability pension during the follow-up period but pass on to old-age pension, emigrate or die before the year 2000, might be incomplete and disability pensioners in sheltered/subsidised jobs are not defined as cases since registration started in 2000. These shortcomings may bias our results slightly towards unity but they are unlikely to change the conclusions. Restricting the analysis to male employees below the age of 50 did not alter the magnitude of the estimates appreciably.

We did not find other estimates of the population attributable fraction so our results need to be confirmed in other studies. The fraction of workers exposed is decreasing especially in agriculture and driving so the estimate may go down in the future. Also future studies aiming to reduce WBV among the exposed are needed.

It may be possible through technical measures and a better organization of work tasks to reduce WBV. This study suggests that a reduction of the incidence of disability pensions could be obtainable through such measures.

## Conclusions

Exposure to WBV predicts subsequent disability pension retirement. Reduction of WBV may reduce the new cases of disability pension.

## Abbreviations

DWECS: Danish Work Environment Cohort Study; DREAM: an administrative register holding weekly information of social transfer payments for all inhabitants in Denmark (DREAM is a Danish acronym for The Register-based Evaluation of Marginalisation); WBV: (Whole-body-vibrations), HR: Hazard ratio; Exp.: Exposed.

## Competing interests

The authors declare that they have no competing interests.

## Authors' contributions

HF, KBC designed the record linkage and carried out the statistical analysis. All authors contributed to the design and discussion of the results. KBC and HF drafted the methods section. FT made the first draft of the other sections. All authors contributed to the text, and they read and approved the final manuscript.

## Pre-publication history

The pre-publication history for this paper can be accessed here:

http://www.biomedcentral.com/1471-2458/10/305/prepub

## References

[B1] Hannerz H Tüchsen F Spangenberg S Albertsen K Industrial differences in disability retirement rates in Denmark 1996 - 2000 International Journal of Occupational Medicine and Environmental Health 2004 17 465 471 15852761

[B2] Albertsen K Lund T Christensen KB Kristensen TS Villadsen E Predictors of disability pension over a 10-year period for men and women Scand J Publ Health 2007 35 78 85 10.1080/14034940600858474 17366091

[B3] Miranda H Viikari-Juntura E Punnett L Riihimaki H Occupational loading, health behavior and sleep disturbance as predictors of low-back pain Scand J Work Environ Health 2008 34 6 411 9 19137202 10.5271/sjweh.1290

[B4] Punnett L Pruss-Utun A Nelson DI Fingerhut MA Leigh J Tak S Phillips S Estimating the global burden of low back pain attributable to combined occupational exposures Am J Ind Med 2005 48 459 469 10.1002/ajim.20232 16299708

[B5] Boshuizen HC Hulshof CT Bongers PM Long-term sick leave and disability pensioning due to back disorders of tractor drivers exposed to whole-body vibration Int Arch Occup Environ Health 1990 62 117 122 10.1007/BF00383587 2139013

[B6] Rehn B Nilsson T Järvholm B Neuromusculoskeletal disorders in the neck and upper extremities among drivers of all-terrain vehicles--a case series BMC Musculoskelet Disord 2004 5 1 10.1186/1471-2474-5-1 14718063 PMC324409

[B7] Nakashima AM Borland MJ Abel SM Measurement of noise and vibration in Canadian forces armoured vehicles Ind Health 2007 45 318 327 10.2486/indhealth.45.318 17485877

[B8] Palmer KT Griffin MJ Bendall H Pannett B Coggon D Prevalence and pattern of occupational exposure to whole body vibration in Great Britain: findings from a national survey Occup Environ Med 2000 57 229 236 10.1136/oem.57.4.229 10810108 PMC1739944

[B9] Hilfert R Köhne G Toussaint R Zerlett G Probleme der ganzkörperschwingungsbelastung von erdbaumaschinenführern Zbl Arbeitsmed 1981 31 199 206 7303990

[B10] Bongers PM Boshuizen HC Hulshof CT Koemeester AP Back disorders in crane operators exposed to whole-body vibration Int Arch Occup Environ Health 1988 60 129 137 10.1007/BF00381494 2964414

[B11] Jensen A Kærlev L Tüchsen F Hannerz H Dahl S Nielsen PS Olsen J Locomotor diseases among male long haul truck drivers and other professional drivers Int Arch Occup Environ Health 2008 81 821 827 10.1007/s00420-007-0270-4 17924129

[B12] Xu Y Bach E Ørhede E Work environment and low-back pain. The influence of occupational activities Occup Environ Med 1997 741 745 10.1136/oem.54.10.741 9404322 PMC1128929

[B13] Palmer KT Griffin MJ Syddall HE Pannett B Cooper C Coggon D The relative importance of whole body vibration and occupational lifting as risk factors for low-back pain Occup Environ Med 2003 60 715 721 10.1136/oem.60.10.715 14504358 PMC1740390

[B14] Messing K Tissot F Stock S Distal lower-extremity pain and work postures in the Quebec population Am J Public Health 2008 98 705 713 10.2105/AJPH.2006.099317 17761561 PMC2377000

[B15] Bongers PM Boshuizen HC Hulshof CT Koemeester AP Long-term sickness absence due to back disorders in crane operators exposed to whole-body vibration Int Arch Occup Environ Health 1988 61 59 64 10.1007/BF00381608 2974020

[B16] Hjollund NH Larsen FB Andersen JH Register-based follow-up of social benefits and other transfer payments: accuracy and degree of completeness in a Danish interdepartmental administrative database compared with a population-based survey Scand J Public Health 2007 35 497 502 10.1080/14034940701271882 17852980

[B17] Burr H Bjorner JB Kristensen TS Tüchsen F Bach E Trends in the Danish work environment in 1990-2000 and their associations with labor-force changes Scand J Work Environ Health 2003 29 270 279 12934720 10.5271/sjweh.731

[B18] Miettinen OS Proportion of disease caused or prevented by a given exposure or intervention Am J Epidemiol 1974 99 325 332 4825599 10.1093/oxfordjournals.aje.a121617

[B19] Statistics Denmark DISCO-88, Statistics Denmark's Standard Classification of Occupations 1996 Copenhagen: Statistics Denmark

[B20] Statistics Denmark Danish Industrial Classification of All Economic Activities 1993 1996 Copenhagen: Statistics Denmark

